# Crystal structure of 2,2′-{[(2-nitro­benz­yl)aza­nedi­yl]bis­(propane-3,1-di­yl)}bis­[1*H*-iso­indole-1,3(2*H*)-dione]

**DOI:** 10.1107/S2056989020016771

**Published:** 2021-01-08

**Authors:** Ryne Holmberg, Vanessa Franz, Kristen M. Moser, Ricardo Solano, Curtis Moore, Arnold L. Rheingold, Gary L. N. Smith

**Affiliations:** a Department of Chemistry, Point Loma Nazarene University, San Diego, CA 92106, USA; b Crystallography Facility, The Ohio State University, Columbus, OH 43210, USA; c Department of Chemistry, University of California-San Diego, La Jolla, CA 92093, USA; d Department of Chemistry, San Diego Miramar College, San Diego, CA 92126, USA

**Keywords:** crystal structure, phthalimides, π–π inter­actions, tripodal ligand

## Abstract

The structure of the title compound, C_29_H_26_N_4_O_6_, exhibits a folded conformation with the three arms all on the same side of the tertiary N atom. The two phthalimide units make a dihedral angle of 12.18 (12)° and the dihedral angles between the benzyl plane and the phthalimide units are 68.08 (7) and 67.71 (7)°. The crystal packing features π–π inter­actions.

## Abstract 2

The structure of the title compound exhibits a folded conformation with the three arms all on the same side of the tertiary N atom. The crystal packing features π–π inter­actions.

## Chemical context   

The coordination chemistry of tripodal tetra­mine ligands has been reviewed and includes structures with pendant arms that are symmetric or asymmetric with respect to the presence of aliphatic and aromatic donor atoms (Blackman, 2005 ▸). The ligands coordinate transition metals or lanthanide ions using all four nitro­gen donor atoms. Tripodal amines have also been shown to coordinate to anions (Bose *et al.*, 2011 ▸; Bazzicalupi *et al.*, 2009 ▸; Kuswandi *et al.*, 2006 ▸). The title compound is an inter­mediate for the synthesis of an asymmetrical tripodal tetra­mine. After removal of the phthalimide protecting groups and reduction of the nitro group, the title compound will become a tripodal ligand with two arms that contain aliphatic nitro­gens and one with an aromatic nitro­gen (Keypour *et al.*, 2008*a* ▸,*b* ▸). Phthalimide compounds are of inter­est themselves because they have the tendency to exhibit a variety of supra­molecular inter­actions in the solid state. These include *n*–π, π–π, dipole–dipole, hydrogen bonding, and other supra­molecular inter­actions (Howell *et al.*, 2003 ▸; Barrett *et al.*, 1995 ▸).
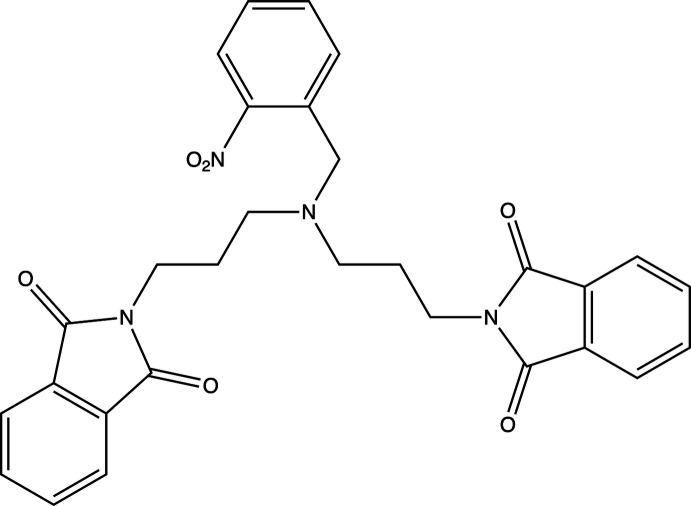


## Structural commentary   

**Figure 1:**
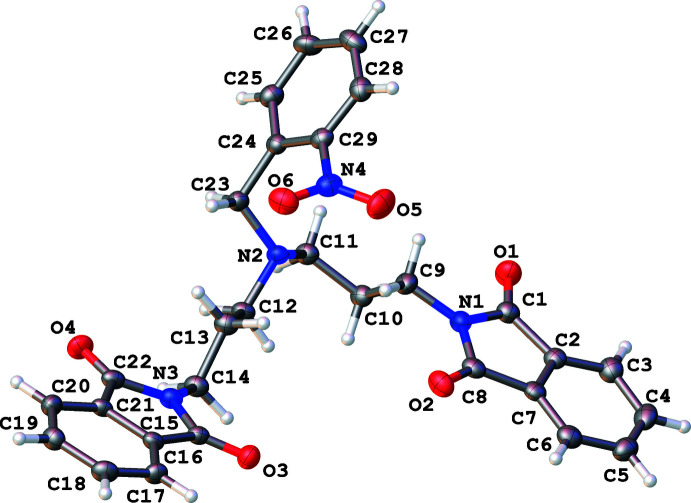
The mol­ecular structure of the title compound, showing 50% probability ellipsoids.

In the title compound (Fig. 1 ▸), the planes of the two phthalimide units (N1/C1–C8 and N3/C15–C22) make a dihedral angle of 12.18 (12)°. The dihedral angles between the benzyl plane and the phthalimide units are 68.08 (7) and 67.71 (7)°. This orientation creates a cavity around which the three arms are arranged. The bridgehead nitro­gen (N2) is located 2.104 (2) Å away from the plane created by the other three nitro­gen atoms.

## Supra­molecular features   

**Figure 2:**
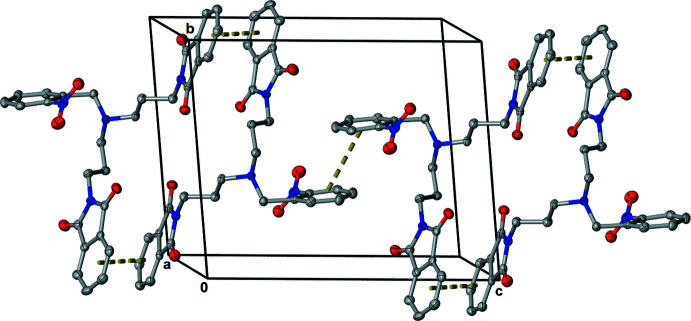
Mol­ecular packing of the title compound showing the π–π inter­actions (dashed lines).

The crystal structure consists of centrosymmetrical dimers with off-set π–π stacking between phthalimide groups (N3/C15–C22) running along the *c*-axis direction (Fig. 2 ▸). The centroid–centroid separation is 3.631 (4) Å. A second π–π stacking inter­action is found with one of the arms. The C*g*(N1/C1–C8)⋯C*g*(N3/C15–C22) centroid–centroid distance is 3.576 (4) Å. There is also a longer centrosymmetric interaction of the nitro benzyl groups (N4/C24–C29) with a distance of 4.694 (5) Å.

## Database survey   

A search of the Cambridge Structural Database (version 5.41, update of October 2020; Groom *et al.*, 2016 ▸) for related compounds with a phthalimide unit gave 2623 hits. A search for the skeletal structure of N(CH_2_CH_2_CH_2_N)_3_ resulted in 149 entries. Similar off-set π–π stacking was seen in another compound with two phthalimide groups (REVYUM; Barrett *et al.*, 1995 ▸). However, it was shown that an intra­molecular hydrogen bond between phthalimide groups resulted in no π–π stacking (VEHRUW; Brycki *et al.*, 2006 ▸). More recently, a urea compound with two phthalimides showed π–π stacking and intra­molecular hydrogen bonding (PONZEZ; Medrano *et al.*, 2014 ▸). Three structures with only one phthalimide group have also shown π–π inter­actions (VIDTUA; Brovarets *et al.*, 2018 ▸; PAVHUR; Yang *et al.*, 2012 ▸; SAGTIF; Shao *et al.*, 2012 ▸). Another compound has been reported that has two phthalimide-protected nitro­gens with two carbon spacers *versus* three for the title compound, a benzyl group, and a trityl sulfide (WOJSIZ; Flörke *et al.*, 2014 ▸). The dihedral angle between the planes of the phthalimide units is significantly different from the title compound at 77.86 (3)°. The crystal packing of this structure shows hydrogen bonding but not π–π stacking.

## Synthesis and crystallization   

The title compound was prepared by using a previously reported method (Keypour *et al.*, 2008 ▸ *a*). 3,3′-Diphthal­imido­di­propyl­amine (5.0 g, 13 mmol), 2-nitro­benzyl­chloride (2.6 g, 15 mmol), and potassium carbonate (1.8 g, 13 mmol) were heated at 433 K for one h to give the title compound. Crystals suitable for X-ray analysis were slowly grown from chloro­form.

## Refinement   

Crystal data, data collection and structure refinement details are summarized in Table 1 ▸. H atoms were positioned geom­etrically (C—H = 0.95–0.99 Å) and refined using a riding model with *U*_iso_(H) = 1.2*U*_eq_(C).

## Supplementary Material

Crystal structure: contains datablock(s) I. DOI: 10.1107/S2056989020016771/zn2003sup1.cif

Supporting information file. DOI: 10.1107/S2056989020016771/zn2003Isup4.cdx

Supporting information file. DOI: 10.1107/S2056989020016771/zn2003Isup3.cml

CCDC reference: 2052908

Additional supporting information:  crystallographic information; 3D view; checkCIF report

## Figures and Tables

**Table 1:** Experimental details

Crystal data	
Chemical formula	C_29_H_26_N_4_O_6_
*M*_r_	526.54
Crystal system, space group	Triclinic, *P* 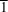
Temperature (K)	120
*a*, *b*, *c* (Å)	7.8576 (10), 12.3468 (15), 14.1147 (17)
α, β, γ (°)	94.295 (1), 104.603 (1), 101.042 (1)
*V* (Å^3^)	1289.6 (3)
*Z*	2
Radiation type	Mo *K*α
μ (mm^−1^)	0.10
Crystal size (mm)	0.15 × 0.05 × 0.01

Data collection	
Diffractometer	Bruker *APEX* CCD
Absorption correction	Multi-scan (*SADABS*; Krause *et al.*, 2015 ▸)
*T*_min_, *T*_max_	0.986, 0.999
No. of measured, independent and observed [*I* > 2σ(*I*)] reflections	11940, 4518, 3422
*R*_int_	0.037
(sin θ/λ)_max_ (Å^−1^)	0.604

Refinement	
*R*[*F* ^2^ > 2σ(*F* ^2^)], *wR*(*F* ^2^), *S*	0.060, 0.124, 1.05
No. of reflections	4518
No. of parameters	352
H-atom treatment	H-atom parameters constrained
Δρ_max_, Δρ_min_ (e Å^−3^)	0.22, −0.24

Computer programs: *APEX2* and *SAINT* (Bruker, 2014 ▸), and *SHELXS97*, *SHELXL97*, and *SHELXTL* (Sheldrick, 2008 ▸).

## supplementary crystallographic information

### Crystal data

**Table:**

C_29_H_26_N_4_O_6_	*Z* = 2
*M**_r_* = 526.54	*F*(000) = 552
Triclinic, *P*1	*D*_x_ = 1.356 Mg m^−^^3^
*a* = 7.8576 (10) Å	Mo *K*α radiation, λ = 0.71073 Å
*b* = 12.3468 (15) Å	Cell parameters from 4970 reflections
*c* = 14.1147 (17) Å	θ = 2.4–25.4°
α = 94.295 (1)°	µ = 0.10 mm^−^^1^
β = 104.603 (1)°	*T* = 120 K
γ = 101.042 (1)°	Plate, colorless
*V* = 1289.6 (3) Å^3^	0.15 × 0.05 × 0.01 mm

### Data collection

**Table:**

Bruker APEX CCD diffractometer	4518 independent reflections
Radiation source: fine-focus sealed tube	3422 reflections with *I* > 2σ(*I*)
Graphite monochromator	*R*_int_ = 0.037
φ and ω scans	θ_max_ = 25.4°, θ_min_ = 1.5°
Absorption correction: multi-scan (SADABS; Krause *et al.*, 2015)	*h* = −9→9
*T*_min_ = 0.986, *T*_max_ = 0.999	*k* = −14→14
11940 measured reflections	*l* = −16→16

### Refinement

**Table:**

Refinement on *F*^2^	Primary atom site location: structure-invariant direct methods
Least-squares matrix: full	Secondary atom site location: difference Fourier map
*R*[*F*^2^ > 2σ(*F*^2^)] = 0.060	Hydrogen site location: inferred from neighbouring sites
*wR*(*F*^2^) = 0.124	H-atom parameters constrained
*S* = 1.05	*w* = 1/[σ^2^(*F*_o_^2^) + (0.0222*P*)^2^ + 1.5*P*] where *P* = (*F*_o_^2^ + 2*F*_c_^2^)/3
4518 reflections	(Δ/σ)_max_ < 0.001
352 parameters	Δρ_max_ = 0.22 e Å^−^^3^
0 restraints	Δρ_min_ = −0.24 e Å^−^^3^

### Special details

**Table:**

Geometry. All esds (except the esd in the dihedral angle between two l.s. planes) are estimated using the full covariance matrix. The cell esds are taken into account individually in the estimation of esds in distances, angles and torsion angles; correlations between esds in cell parameters are only used when they are defined by crystal symmetry. An approximate (isotropic) treatment of cell esds is used for estimating esds involving l.s. planes.
Refinement. Refinement of F^2^ against ALL reflections. The weighted R-factor wR and goodness of fit S are based on F^2^, conventional R-factors R are based on F, with F set to zero for negative F^2^. The threshold expression of F^2^ > 2sigma(F^2^) is used only for calculating R-factors(gt) etc. and is not relevant to the choice of reflections for refinement. R-factors based on F^2^ are statistically about twice as large as those based on F, and R- factors based on ALL data will be even larger.

### Fractional atomic coordinates and isotropic or equivalent isotropic displacement parameters (Å^2^)

**Table:**

	*x*	*y*	*z*	*U*_iso_*/*U*_eq_	
C1	0.4890 (4)	0.8275 (2)	0.3635 (2)	0.0271 (7)	
C2	0.6627 (4)	0.9044 (2)	0.3687 (2)	0.0243 (6)	
C3	0.7287 (4)	1.0141 (2)	0.4092 (2)	0.0311 (7)	
H3	0.6606	1.0541	0.4398	0.037*	
C4	0.9008 (4)	1.0641 (3)	0.4031 (2)	0.0351 (8)	
H4	0.9497	1.1401	0.4292	0.042*	
C5	1.0010 (4)	1.0052 (3)	0.3599 (2)	0.0337 (8)	
H5	1.1175	1.0414	0.3572	0.040*	
C6	0.9345 (4)	0.8945 (3)	0.3206 (2)	0.0301 (7)	
H6	1.0036	0.8536	0.2916	0.036*	
C7	0.7637 (4)	0.8458 (2)	0.3253 (2)	0.0241 (6)	
C8	0.6567 (4)	0.7305 (2)	0.2896 (2)	0.0245 (6)	
C9	0.3523 (4)	0.6256 (2)	0.2976 (2)	0.0294 (7)	
H9A	0.3190	0.6152	0.3600	0.035*	
H9B	0.3999	0.5606	0.2796	0.035*	
C10	0.1841 (4)	0.6293 (2)	0.2168 (2)	0.0256 (6)	
H10A	0.1377	0.6952	0.2335	0.031*	
H10B	0.2155	0.6367	0.1535	0.031*	
C11	0.0385 (4)	0.5247 (2)	0.2048 (2)	0.0261 (6)	
H11A	−0.0070	0.5243	0.2641	0.031*	
H11B	−0.0633	0.5259	0.1472	0.031*	
C12	0.1262 (3)	0.4048 (2)	0.0910 (2)	0.0237 (6)	
H12A	0.1831	0.4769	0.0743	0.028*	
H12B	0.0056	0.3794	0.0434	0.028*	
C13	0.2396 (4)	0.3205 (2)	0.0790 (2)	0.0264 (7)	
H13A	0.3572	0.3417	0.1297	0.032*	
H13B	0.1775	0.2461	0.0883	0.032*	
C14	0.2694 (4)	0.3165 (2)	−0.0232 (2)	0.0264 (6)	
H14A	0.1517	0.3070	−0.0728	0.032*	
H14B	0.3454	0.3884	−0.0285	0.032*	
C15	0.5367 (3)	0.2414 (2)	−0.0430 (2)	0.0221 (6)	
C16	0.5587 (3)	0.1320 (2)	−0.08339 (19)	0.0206 (6)	
C17	0.7090 (3)	0.0994 (2)	−0.0999 (2)	0.0252 (6)	
H17	0.8233	0.1495	−0.0828	0.030*	
C18	0.6859 (4)	−0.0095 (3)	−0.1424 (2)	0.0286 (7)	
H18	0.7866	−0.0343	−0.1547	0.034*	
C19	0.5197 (4)	−0.0827 (2)	−0.1672 (2)	0.0268 (7)	
H19	0.5079	−0.1564	−0.1969	0.032*	
C20	0.3687 (4)	−0.0497 (2)	−0.1492 (2)	0.0256 (6)	
H20	0.2545	−0.0997	−0.1652	0.031*	
C21	0.3928 (3)	0.0578 (2)	−0.10763 (19)	0.0203 (6)	
C22	0.2602 (3)	0.1187 (2)	−0.0827 (2)	0.0238 (6)	
C23	−0.0218 (3)	0.3273 (2)	0.2100 (2)	0.0238 (6)	
H23A	0.0068	0.2568	0.1872	0.029*	
H23B	−0.1465	0.3279	0.1729	0.029*	
C24	−0.0079 (3)	0.3337 (2)	0.3192 (2)	0.0229 (6)	
C25	−0.1560 (4)	0.3386 (2)	0.3552 (2)	0.0284 (7)	
H25	−0.2697	0.3350	0.3098	0.034*	
C26	−0.1421 (4)	0.3485 (3)	0.4551 (2)	0.0325 (7)	
H26	−0.2470	0.3475	0.4771	0.039*	
C27	0.0227 (4)	0.3597 (3)	0.5233 (2)	0.0337 (7)	
H27	0.0318	0.3680	0.5920	0.040*	
C28	0.1741 (4)	0.3589 (2)	0.4909 (2)	0.0282 (7)	
H28	0.2892	0.3687	0.5368	0.034*	
C29	0.1551 (4)	0.3436 (2)	0.3907 (2)	0.0247 (6)	
N1	0.4939 (3)	0.72652 (19)	0.31379 (17)	0.0256 (6)	
N2	0.1039 (3)	0.42205 (19)	0.19079 (16)	0.0217 (5)	
N3	0.3550 (3)	0.22716 (19)	−0.04578 (17)	0.0227 (5)	
N4	0.3188 (3)	0.3337 (2)	0.36090 (19)	0.0298 (6)	
O1	0.3652 (3)	0.84351 (18)	0.39538 (16)	0.0375 (5)	
O2	0.6965 (3)	0.65364 (18)	0.24824 (15)	0.0328 (5)	
O3	0.6476 (2)	0.32780 (17)	−0.01314 (15)	0.0300 (5)	
O4	0.1007 (2)	0.08489 (17)	−0.09265 (15)	0.0315 (5)	
O5	0.4580 (3)	0.4043 (2)	0.40052 (17)	0.0434 (6)	
O6	0.3096 (3)	0.25346 (19)	0.30131 (17)	0.0373 (5)	

### Atomic displacement parameters (Å^2^)

**Table:**

	*U*^11^	*U*^22^	*U*^33^	*U*^12^	*U*^13^	*U*^23^
C1	0.0285 (15)	0.0301 (18)	0.0236 (16)	0.0078 (13)	0.0073 (12)	0.0052 (13)
C2	0.0283 (14)	0.0226 (16)	0.0236 (16)	0.0086 (12)	0.0065 (11)	0.0071 (12)
C3	0.0363 (16)	0.0261 (18)	0.0316 (18)	0.0104 (14)	0.0071 (13)	0.0060 (14)
C4	0.0416 (18)	0.0235 (17)	0.0344 (19)	0.0016 (14)	0.0021 (14)	0.0095 (14)
C5	0.0295 (16)	0.0357 (19)	0.0338 (18)	0.0034 (14)	0.0059 (13)	0.0110 (15)
C6	0.0314 (15)	0.0358 (19)	0.0242 (16)	0.0113 (14)	0.0054 (12)	0.0076 (14)
C7	0.0290 (14)	0.0267 (17)	0.0179 (15)	0.0086 (12)	0.0055 (11)	0.0069 (12)
C8	0.0311 (15)	0.0264 (17)	0.0195 (15)	0.0127 (13)	0.0072 (11)	0.0069 (13)
C9	0.0335 (16)	0.0239 (17)	0.0293 (17)	0.0019 (13)	0.0086 (12)	0.0060 (13)
C10	0.0308 (15)	0.0202 (16)	0.0281 (16)	0.0076 (12)	0.0106 (12)	0.0035 (13)
C11	0.0295 (15)	0.0248 (17)	0.0275 (16)	0.0095 (12)	0.0113 (12)	0.0047 (13)
C12	0.0248 (14)	0.0283 (17)	0.0215 (15)	0.0091 (12)	0.0092 (11)	0.0066 (13)
C13	0.0275 (15)	0.0277 (17)	0.0277 (16)	0.0106 (12)	0.0100 (12)	0.0056 (13)
C14	0.0299 (15)	0.0269 (17)	0.0273 (16)	0.0114 (12)	0.0119 (12)	0.0057 (13)
C15	0.0218 (13)	0.0268 (17)	0.0203 (15)	0.0072 (13)	0.0081 (11)	0.0053 (12)
C16	0.0189 (13)	0.0275 (16)	0.0172 (14)	0.0078 (11)	0.0058 (10)	0.0040 (12)
C17	0.0193 (13)	0.0297 (17)	0.0288 (16)	0.0068 (12)	0.0090 (11)	0.0056 (13)
C18	0.0252 (14)	0.0369 (19)	0.0306 (17)	0.0145 (13)	0.0129 (12)	0.0084 (14)
C19	0.0307 (15)	0.0247 (17)	0.0269 (16)	0.0096 (12)	0.0091 (12)	0.0021 (13)
C20	0.0212 (13)	0.0286 (17)	0.0255 (16)	0.0023 (12)	0.0061 (11)	0.0028 (13)
C21	0.0190 (13)	0.0275 (17)	0.0177 (14)	0.0091 (11)	0.0073 (10)	0.0042 (12)
C22	0.0197 (14)	0.0337 (18)	0.0209 (15)	0.0094 (12)	0.0072 (11)	0.0072 (13)
C23	0.0223 (13)	0.0259 (16)	0.0232 (15)	0.0042 (12)	0.0073 (11)	0.0032 (12)
C24	0.0267 (14)	0.0184 (15)	0.0243 (15)	0.0044 (11)	0.0084 (11)	0.0033 (12)
C25	0.0251 (14)	0.0288 (17)	0.0321 (17)	0.0066 (12)	0.0078 (12)	0.0070 (14)
C26	0.0367 (16)	0.0370 (19)	0.0336 (18)	0.0152 (14)	0.0204 (13)	0.0100 (15)
C27	0.0499 (19)	0.0323 (19)	0.0247 (17)	0.0175 (15)	0.0135 (14)	0.0067 (14)
C28	0.0316 (15)	0.0228 (17)	0.0290 (17)	0.0078 (13)	0.0042 (12)	0.0045 (13)
C29	0.0267 (14)	0.0196 (15)	0.0307 (17)	0.0067 (12)	0.0108 (12)	0.0062 (13)
N1	0.0305 (13)	0.0226 (14)	0.0246 (13)	0.0056 (10)	0.0090 (10)	0.0026 (11)
N2	0.0239 (11)	0.0214 (13)	0.0234 (13)	0.0076 (10)	0.0107 (9)	0.0039 (10)
N3	0.0236 (12)	0.0243 (14)	0.0245 (13)	0.0104 (10)	0.0098 (9)	0.0045 (10)
N4	0.0275 (13)	0.0311 (16)	0.0341 (15)	0.0100 (12)	0.0096 (11)	0.0109 (13)
O1	0.0348 (12)	0.0385 (14)	0.0422 (14)	0.0066 (10)	0.0193 (10)	−0.0035 (11)
O2	0.0415 (12)	0.0304 (13)	0.0296 (12)	0.0160 (10)	0.0103 (9)	0.0012 (10)
O3	0.0278 (10)	0.0280 (12)	0.0334 (12)	0.0031 (9)	0.0098 (9)	0.0008 (9)
O4	0.0189 (10)	0.0399 (13)	0.0379 (13)	0.0071 (9)	0.0114 (8)	0.0042 (10)
O5	0.0249 (11)	0.0481 (15)	0.0528 (15)	0.0032 (10)	0.0049 (10)	0.0100 (12)
O6	0.0431 (13)	0.0379 (14)	0.0391 (14)	0.0202 (11)	0.0168 (10)	0.0063 (11)

### Geometric parameters (Å, º)

**Table:**

C1—O1	1.212 (3)	C14—H14B	0.9900
C1—N1	1.396 (4)	C15—O3	1.211 (3)
C1—C2	1.487 (4)	C15—N3	1.394 (3)
C2—C3	1.377 (4)	C15—C16	1.485 (4)
C2—C7	1.388 (4)	C16—C17	1.385 (4)
C3—C4	1.401 (4)	C16—C21	1.388 (4)
C3—H3	0.9500	C17—C18	1.390 (4)
C4—C5	1.384 (4)	C17—H17	0.9500
C4—H4	0.9500	C18—C19	1.384 (4)
C5—C6	1.384 (4)	C18—H18	0.9500
C5—H5	0.9500	C19—C20	1.400 (4)
C6—C7	1.382 (4)	C19—H19	0.9500
C6—H6	0.9500	C20—C21	1.369 (4)
C7—C8	1.486 (4)	C20—H20	0.9500
C8—O2	1.209 (3)	C21—C22	1.490 (4)
C8—N1	1.398 (4)	C22—O4	1.211 (3)
C9—N1	1.464 (3)	C22—N3	1.392 (4)
C9—C10	1.524 (4)	C23—N2	1.469 (3)
C9—H9A	0.9900	C23—C24	1.512 (4)
C9—H9B	0.9900	C23—H23A	0.9900
C10—C11	1.520 (4)	C23—H23B	0.9900
C10—H10A	0.9900	C24—C25	1.391 (4)
C10—H10B	0.9900	C24—C29	1.394 (4)
C11—N2	1.474 (3)	C25—C26	1.381 (4)
C11—H11A	0.9900	C25—H25	0.9500
C11—H11B	0.9900	C26—C27	1.380 (4)
C12—N2	1.467 (3)	C26—H26	0.9500
C12—C13	1.520 (4)	C27—C28	1.379 (4)
C12—H12A	0.9900	C27—H27	0.9500
C12—H12B	0.9900	C28—C29	1.379 (4)
C13—C14	1.517 (4)	C28—H28	0.9500
C13—H13A	0.9900	C29—N4	1.474 (4)
C13—H13B	0.9900	N4—O6	1.229 (3)
C14—N3	1.452 (3)	N4—O5	1.234 (3)
C14—H14A	0.9900		
			
O1—C1—N1	124.5 (3)	O3—C15—N3	124.6 (3)
O1—C1—C2	129.7 (3)	O3—C15—C16	129.6 (2)
N1—C1—C2	105.8 (2)	N3—C15—C16	105.9 (2)
C3—C2—C7	121.3 (3)	C17—C16—C21	120.9 (3)
C3—C2—C1	130.6 (3)	C17—C16—C15	130.6 (2)
C7—C2—C1	108.1 (2)	C21—C16—C15	108.5 (2)
C2—C3—C4	117.0 (3)	C16—C17—C18	117.3 (3)
C2—C3—H3	121.5	C16—C17—H17	121.3
C4—C3—H3	121.5	C18—C17—H17	121.3
C5—C4—C3	121.5 (3)	C19—C18—C17	121.5 (3)
C5—C4—H4	119.3	C19—C18—H18	119.2
C3—C4—H4	119.3	C17—C18—H18	119.2
C6—C5—C4	121.2 (3)	C18—C19—C20	120.9 (3)
C6—C5—H5	119.4	C18—C19—H19	119.5
C4—C5—H5	119.4	C20—C19—H19	119.5
C7—C6—C5	117.3 (3)	C21—C20—C19	117.1 (2)
C7—C6—H6	121.3	C21—C20—H20	121.5
C5—C6—H6	121.3	C19—C20—H20	121.5
C6—C7—C2	121.8 (3)	C20—C21—C16	122.3 (2)
C6—C7—C8	129.9 (3)	C20—C21—C22	130.1 (2)
C2—C7—C8	108.4 (2)	C16—C21—C22	107.5 (2)
O2—C8—N1	125.2 (3)	O4—C22—N3	124.9 (3)
O2—C8—C7	129.1 (3)	O4—C22—C21	128.9 (3)
N1—C8—C7	105.7 (2)	N3—C22—C21	106.3 (2)
N1—C9—C10	112.9 (2)	N2—C23—C24	110.2 (2)
N1—C9—H9A	109.0	N2—C23—H23A	109.6
C10—C9—H9A	109.0	C24—C23—H23A	109.6
N1—C9—H9B	109.0	N2—C23—H23B	109.6
C10—C9—H9B	109.0	C24—C23—H23B	109.6
H9A—C9—H9B	107.8	H23A—C23—H23B	108.1
C11—C10—C9	111.1 (2)	C25—C24—C29	115.5 (3)
C11—C10—H10A	109.4	C25—C24—C23	121.7 (2)
C9—C10—H10A	109.4	C29—C24—C23	122.6 (2)
C11—C10—H10B	109.4	C26—C25—C24	121.7 (3)
C9—C10—H10B	109.4	C26—C25—H25	119.1
H10A—C10—H10B	108.0	C24—C25—H25	119.1
N2—C11—C10	112.6 (2)	C27—C26—C25	120.7 (3)
N2—C11—H11A	109.1	C27—C26—H26	119.7
C10—C11—H11A	109.1	C25—C26—H26	119.7
N2—C11—H11B	109.1	C28—C27—C26	119.4 (3)
C10—C11—H11B	109.1	C28—C27—H27	120.3
H11A—C11—H11B	107.8	C26—C27—H27	120.3
N2—C12—C13	113.8 (2)	C27—C28—C29	118.7 (3)
N2—C12—H12A	108.8	C27—C28—H28	120.6
C13—C12—H12A	108.8	C29—C28—H28	120.6
N2—C12—H12B	108.8	C28—C29—C24	123.8 (3)
C13—C12—H12B	108.8	C28—C29—N4	116.1 (2)
H12A—C12—H12B	107.7	C24—C29—N4	120.1 (3)
C14—C13—C12	109.8 (2)	C1—N1—C8	112.1 (2)
C14—C13—H13A	109.7	C1—N1—C9	123.7 (2)
C12—C13—H13A	109.7	C8—N1—C9	124.0 (2)
C14—C13—H13B	109.7	C12—N2—C23	111.5 (2)
C12—C13—H13B	109.7	C12—N2—C11	110.5 (2)
H13A—C13—H13B	108.2	C23—N2—C11	109.4 (2)
N3—C14—C13	113.3 (2)	C22—N3—C15	111.8 (2)
N3—C14—H14A	108.9	C22—N3—C14	123.3 (2)
C13—C14—H14A	108.9	C15—N3—C14	124.5 (2)
N3—C14—H14B	108.9	O6—N4—O5	124.3 (3)
C13—C14—H14B	108.9	O6—N4—C29	118.0 (2)
H14A—C14—H14B	107.7	O5—N4—C29	117.7 (3)
